# BioSAVE: Display of scored annotation within a sequence context

**DOI:** 10.1186/1471-2105-9-157

**Published:** 2008-03-20

**Authors:** Richard F Pollock, Boris Adryan

**Affiliations:** 1 MRC Laboratory of Molecular Biology, Hills Road, Cambridge, CB2 0QH, UK

## Abstract

**Background:**

Visualization of sequence annotation is a common feature in many bioinformatics tools. For many applications it is desirable to restrict the display of such annotation according to a score cutoff, as biological interpretation can be difficult in the presence of the entire data. Unfortunately, many visualisation solutions are somewhat static in the way they handle such score cutoffs.

**Results:**

We present BioSAVE, a sequence annotation viewer with on-the-fly selection of visualisation thresholds for each feature. BioSAVE is a versatile OS X program for visual display of scored features (annotation) within a sequence context. The program reads sequence and additional supplementary annotation data (e.g., position weight matrix matches, conservation scores, structural domains) from a variety of commonly used file formats and displays them graphically. Onscreen controls then allow for live customisation of these graphics, including on-the-fly selection of visualisation thresholds for each feature.

**Conclusion:**

Possible applications of the program include display of transcription factor binding sites in a genomic context or the visualisation of structural domain assignments in protein sequences and many more. The dynamic visualisation of these annotations is useful, e.g., for the determination of cutoff values of predicted features to match experimental data. Program, source code and exemplary files are freely available at the BioSAVE homepage.

## Background

Visualization of sequence annotation is a common feature in many bioinformatics tools. For example, the widely used UCSC Genome Browser [1] and Ensembl [2] web sites, and also standalone programs such as Artemis [3] or the Integrated Genome Browser [4] graphically display genetic features in their genomic context. These tools also provide means to import additional user-defined numerical annotation, e.g., for the display of experimental data along the genome. These data are then displayed either as continuous line or bar graphs along the sequence, or as boxes covering a sequence range coloured according to the numerical data, i.e., a scored annotation.

A better understanding of a data set can often be achieved when its complexity can be reduced. While all of the aforementioned frameworks are suited to display user-defined data in its entirety, they do not allow for convenient and dynamic customisation of the graphics on-the-fly. The genome browsers are similar to other previously published tools for visualising annotation, such as gff2ps [5], in that they produce "static" output as either a Web page or a PostScript file. The Artemis and Integrated Genome Browser programs have their strength in presenting gene annotation or tiling array data, but are not easily customisable for display of other data types such as position weight matrix matches.

## Implementation

Here, we present BioSAVE (from Biological Sequence Annotation Visualisation), a graphical Mac OS X application for visualisation of scored annotation in a sequence context. It is written in Objective C and makes use of OS X-specific system libraries for responsive display of annotation changes. A plug-in interface allows to invoke external scripts (e.g., written in Perl or Python) upon loading an annotation file, which is useful for converting external formats into GFF2.

## Results and Discussion

BioSAVE will read sequence data in FASTA format [5] or raw text, annotation scores from GFF2 files [6] or, using a plug-in interface, any other parse-able file format that contains coordinates and scores for an annotation. Only data with scores that lie within a user-specified score range will then be displayed along the sequence. Adjusting the score thresholds will dynamically change which annotations are visible, and at which colour level. Where other tools only support a fixed score range (e.g., 0..1000), this range automatically adjusts to the data present in the user's input. In addition to the dynamic view, BioSAVE can display additional tracks along the sequence comprising a conventional bar graph visualisation. The sequence view itself can be adjusted and allows for continuous zooming from the entire sequence down to the nucleotide level. In our hands, on a 1.67 GHz PowerPC G4 with 1 GB of RAM, BioSAVE can reliably handle up to 3 Mb of sequence along with about 3,000 microarray data points for numerical annotation.

The versatility of BioSAVE extends to the display of annotation scores also in protein sequences. Only a small number of tools for handling protein sequence allow to load and display additional numerical annotation (e.g., the Utopia Toolset [7]). However, these programs are comparable to the static display of Artemis and the Integrated Genome Browser, and do not allow for the on-the-fly customisation found in BioSAVE.

In the following section, we discuss two applications in which BioSAVE could be used in day-to-day research.

### Usage example: Transcription factor binding site matches in a DNA sequence

The binding specificity of a transcription factor (TF) or other DNA-binding protein is often represented as position weight matrix (PWM), which allows for a numerical description of the binding site rather than a fixed search string. Many theoretical frameworks and bioinformatic tools exists to identify nucleotide sequences that are matched by a PWM, e.g., Patser [8], Clover [9] and Motifscanner [10]. While these programs are powerful in the identification of PWM matches to a sequence, they all lack an immediate and intuitive display of the results, which is key to make useful biological interpretations.

The difficulty in the interpretation of PWM matches to a sequence is often the lack of a score threshold that reliably distinguishes biologically relevant matches from closely related hits in the sequence. Thus, it is desirable to display the identified PWM matches at various score cutoffs. This helps to establish a cutoff at which the PWM match is convincing, both in terms of its similarity with the sequence, and, if known, whether biologically relevant sequences are being recognised. For Patser and Clover, the Web interfaces RSA-tools [11] and MotifViz [12] are available to visualize PWM matches in input sequences, thus making the results accessible to the bench biologist. However, by default, these interfaces display every possible match to the PWM and lack the ability to dynamically adjust score thresholds.

**Figure 1:**
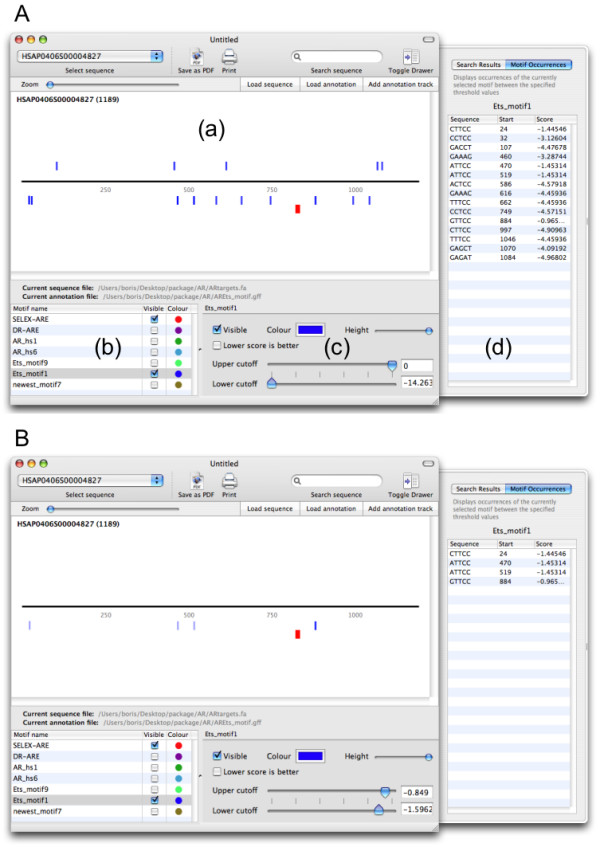
**Visualisation of putative transcription factor binding sites in BioSAVE**. **(A) **This screenshot displays an overview of a 1.2 kb sequence that was shown to contain functional binding sites for the physically interacting androgen receptor (AR) and Ets transcription factors. Matches to the position weight matrices (displayed in **(a)**) of both factors were identified using the Motifscanner tool from the NestedMICA package. After selection in **(b) **they are being displayed as red (AR) or blue (Ets) bars above (+) or below (-) the sequence. Using the selected score threshold shown in **(c)**, there is a wealth of putative Ets sites, as can be seen in the sequence view or motif occurrences table showing corresponding sites **(d)**. **(B) **The score thresholds for the Ets factors are now adjusted to allow only the strongest putative binding sites to be shown, helping the biologist to identify biologically relevant matches to the position weight matrix.

Figure 1 shows how BioSAVE can be used to assess a wealth of putative binding sites. In this example, the presence of a binding site for the androgen receptor (AR) was established experimentally, and it is known that AR physically interacts with the Ets TF [13]. However, the PWM for Ets is short and therefore produces many matches to the sequence. By adjusting the score cutoffs in BioSAVE, the most promising candidate Ets site for experimental validation can quickly be visually identified, both by reducing the number of motifs and graphically displaying the quality of match as a colour code.

**Figure 2:**
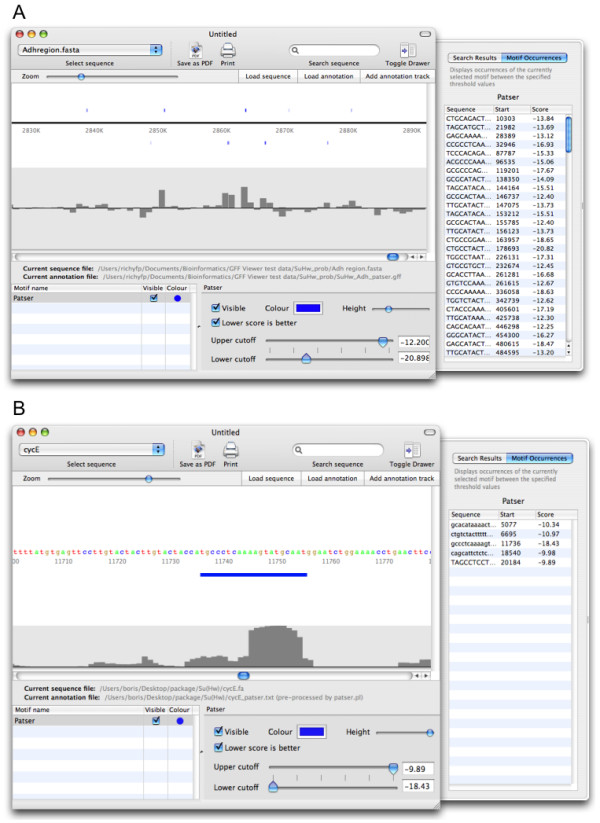
**Combining different sources of information to visually assess putative transcription factor binding sites**. **(A) **The sequence view shows matches to the position weight matrix of the Su(Hw) insulator protein (blue bars) in a portion of the 3 Mb *Adh *region of *D. melanogaster*. The validity of these matches is supported by a chromatin immunoprecipitation (ChIP) experiment with microarray detection. The track underneath the sequence view shows ChIP enrichment for Su(Hw) along a 1 kb tiling path. **(B) **On the nucleotide level, Su(Hw) sites show a good degree of conservation although they are generally located in lowly conserved regions. Sequence conservation is here displayed using phastCon scores. The Su(Hw) position weight matrix matches were generated with Patser and loaded through the BioSAVE plug-in system; the phastCon scores were supplied as GFF file.

A common problem in the interpretation of chromatin immunoprecipitation (ChIP) experiments is to precisely identify the TF binding site (usually comprising a few nucleotides) from a much larger region showing ChIP enrichment for the factor. In Figure 2, a portion of the *Adh *region in the *D. melanogaster *genome and the results of a ChIP experiment for the Suppressor of Hairy Wing (Su(Hw)) protein [14] are being shown. BioSAVE shows a clear correlation between ChIP enrichment along a 1 kb tiling path and the strength of Su(Hw) PWM matches (here, the PWM is indeed a good predictor). At the nucleotide level, focussing on the *cyclin E *gene within the *Adh *region, a validated binding site is shown alongside phastCon scores [15], which further characterise it in terms of evolutionary conservation.

### Usage example: Structural hidden Markov model matches in a protein sequence

**Figure 3:**
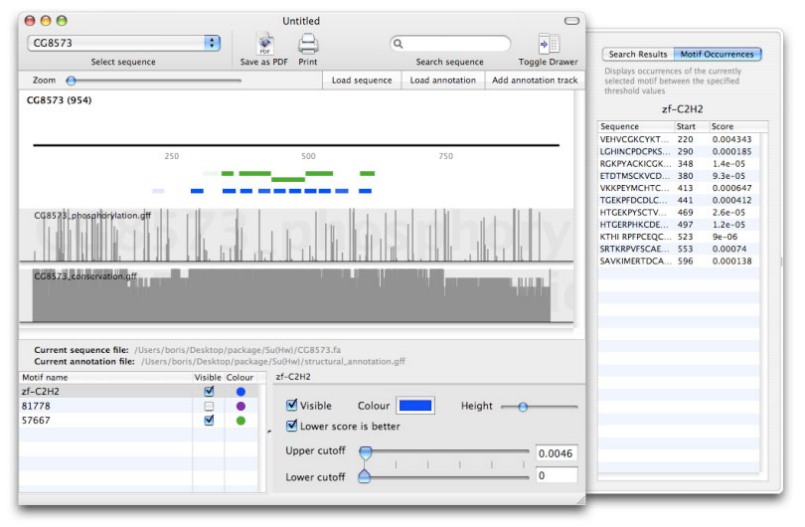
**Visualisation of structural annotation in a protein sequence**. The sequence view shows the primary sequence of the Su(Hw) protein, along with matches to hidden Markov models identifying zinc finger domains according to the Pfam (blue) and Superfamily (green) databases. The two additional tracks show the score of predicted protein phosphorylation sites from the NetPhos 2.0 server and an arbitrary score based on sequence identity within six closely related *Drosophila *species.

Functional annotation of proteins includes the assignment of structural domains. A common strategy is to identify regions of homology to functionally characterised domains. This is often achieved by searching the protein sequence with hidden Markov models from databases such as Superfamily [16] and Pfam [17]. Figure 3 shows the structural annotation of the Su(Hw) protein, along with data of putative phosphorylation sites from the NetPhos 2.0 server [18] and the degree of sequence identity seen amongst six closely related *Drosophila *species. It can be observed that phosphorylation sites are relatively sparse within the predicted zinc finger domains, which are evolutionary highly conserved in this protein.

## Conclusion

BioSAVE is a highly focused, easy-to-use tool for viewing scored annotation in a sequence context. It has many advantages over other sequence annotation visualisation systems not specifically designed for this task. For example, the aforementioned Web interfaces do not allow for prompt user interaction, and other tools developed primarily for visualisation of microarray data are too inflexible to handle scored annotation of different types or sources appropriately, e.g., quick highlighting of matches to a particular PWM etc. To our knowledge, BioSAVE is the only tool for annotation visualisation that handles DNA and protein sequences alike.

This paper describes several applications in which BioSAVE is used in our day-to-day research. Through its support of standard sequence and annotation formats, as well as the versatile plug-in system (employing any script or program that outputs GFF format), BioSAVE can be used in a wide range of applications not specifically discussed here. For example, it can be used for display of mutation hotspots in DNA or protein sequences (e.g., showing frequency) or display of predicted interaction surfaces along a protein sequence (e.g., showing a p-value).

## Availability and requirements

The universal binary for Mac OS X is available for download at the BioSAVE web site [19], along with an extensive tutorial detailing many features not previously mentioned in this paper. The Objective C source code is available from the web site under the GNU GPL.

Project name: BioSAVE

Home page: 

Operating system: OS X

Programming language: Objective C

Other requirements: None

License: Freely available, source code under GNU GPL.

## List of abbreviations

GFF – general feature format, TF – transcription factor, PWM – position weight matrix

## Authors' contributions

RFP wrote the software and created the web site. BA defined the software architecture and created demonstration and test datasets. Both authors wrote the manuscript.
